# Wild sea otter mussel pounding leaves archaeological traces

**DOI:** 10.1038/s41598-019-39902-y

**Published:** 2019-03-14

**Authors:** Michael Haslam, Jessica Fujii, Sarah Espinosa, Karl Mayer, Katherine Ralls, M. Tim Tinker, Natalie Uomini

**Affiliations:** 1 Independent Researcher, Putney Bridge Road, London, SW15 2PA UK; 2 Monterey Bay Aquarium, 886 Cannery Row, Monterey, CA 93940 USA; 3 Department of Ecology and Evolutionary Biology, University of California, Santa Cruz, Long Marine Lab, 115 McAllister Way, Santa Cruz, CA 95060 USA; 4 Center for Conservation Genomics, Smithsonian Conservation Biology Institute, National Zoological Park, 3001 Connecticut Avenue NW, Washington, DC 20008 USA; 5 Nhydra Ecological Consulting, 11 Parklea Dr., Head of St Margarets Bay, Nova Scotia, B3Z 2G6 Canada; 6 Department of Linguistic and Cultural Evolution, Max Planck Institute for the Science of Human History, Kahlaische Strasse 10, 07745 Jena, Germany

## Abstract

Wild sea otters (*Enhydra lutris*) are the only marine mammals that habitually use stones while foraging, using them to break open hard-shelled foods like marine snails and bivalves. However, the physical effects of this behavior on local environments are unknown. We show that sea otters pounding mussels on tidally emergent rocks leave distinct material traces, which can be recognized using methods from archaeology. We observed sea otters pounding mussels at the Bennett Slough Culverts site, California, USA, over a l0-year period. Sea otters repeatedly used the same rocks as anvils, which resulted in distinctive wear patterns on the rocks and accumulations of broken mussel shells, all fractured in a characteristic way, below them. Our results raise the potential for discovery of similar sea otter pounding sites in areas that no longer have resident sea otter populations.

## Introduction

Sea otter (*Enhydra lutris*) populations currently survive only in remnants of their former habitat, which stretched from Baja California, Mexico, around the northern Pacific rim to Japan^1^. Wild sea otters are the only marine mammal known to habitually use stone tools^2^, and they exhibit inter- and intra-population variation in the frequency of tool-use^3,4^. A significantly higher percentage of individuals use tools among the southern sea otters (*E. l. nereis*) than those in the northerly Aleutian Islands, partly due to the hardness of targeted prey: otters use tools less often when consuming soft-bodied prey such as worms than hard-shelled bivalves or marine snails^3,4^. Since stones provide the longest-lasting material evidence of past tool behavior in animals^5–10^, they offer the potential for long-term reconstruction of past sea otter behavior.

**Figure 1:**
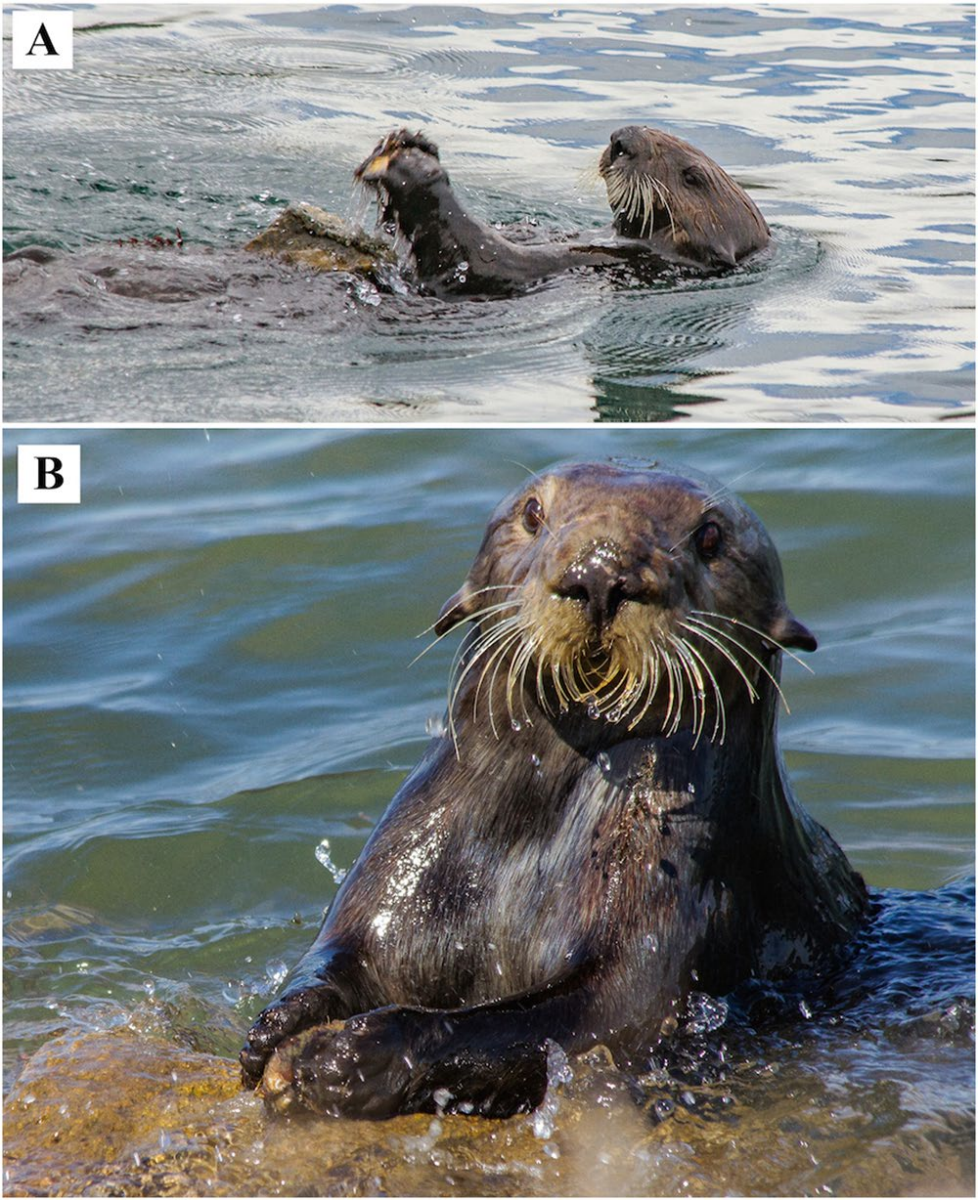
Wild sea otters at Bennett Slough Culverts opening mussels using stones. The otters are using (**A**) a chest anvil, and (**B**) an emergent anvil.

Sea otter stone use while foraging takes three forms: (i) using a stone underwater to pry loose abalone from a substrate^11^, (ii) pounding food using a stone as a hammer or anvil on the chest while floating at the surface^12^ (Fig. 1A), and (iii) pounding food directly against a rocky substrate. Both the underwater and chest anvil pounding behaviors are considered tool-use under current definitions^2^, as they involve the controlled use of a detached object. In the third form of stone use, the sea otter repeatedly pounds a hard-shelled prey against a stationary, fixed stone anvil, typically a boulder at the water margin (Fig. 1B). We term this behavior *emergent anvil* use, to distinguish it from the use of chest anvils. There are no data at present on the selection or rate of re-use of stone tools among sea otters.

**Figure 2:**
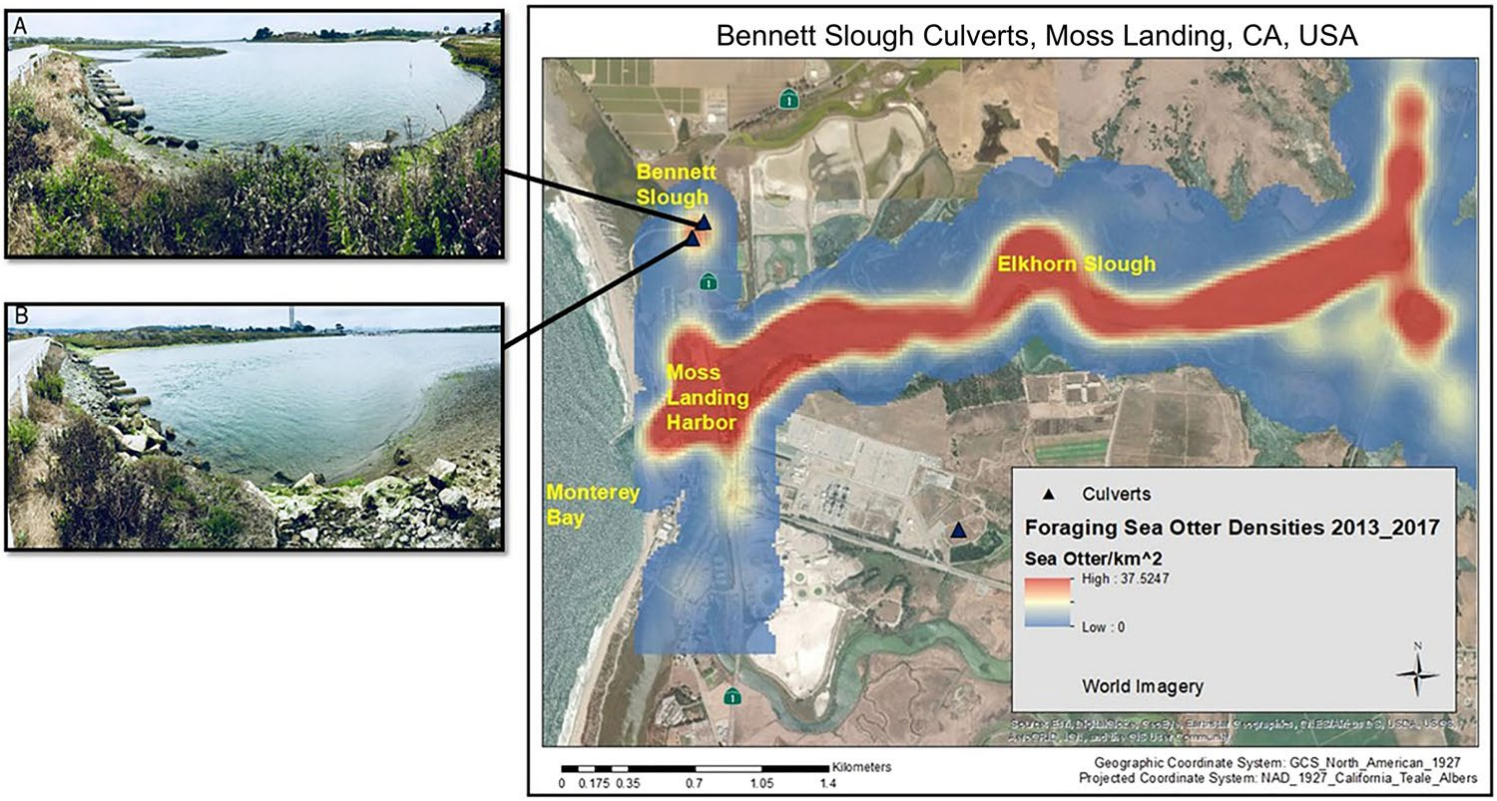
Map of the Bennett Slough Culverts (BSC) study site and Moss Landing, with foraging sea otter densities. Black triangles show the position of BSC North and South, and the insets show (**A**) BSC North facing northwest, and (**B**) BSC South facing southeast. Jetty Road is at the left of both inset photos. The map was created using ArcGIS 10.6.1 (ESRI 2018, Redlands, CA). The kernel density of foraging sea otters was created using the Spatial Analyst toolbox on sea otter location data from distribution surveys from January to December 2016. Kernel densities in raster format were calculated using a grid cell size of 400 m^2^ and a kernel-smoothing window of 200 m. Kernel density is displayed with a transparency of 30% to see the features of Moss Landing on the ESRI World Imagery Basemap (Sources: Esri, DigitalGlobe, Earthstar Geographics, CNES/Airbus DS, GeoEye, USDA FSA, USGS, Aerogrid, IGN, IGP, and the GIS User Community, https://services.arcgisonline.com/ArcGIS/rest/services/World_Imagery/MapServer).

**Figure 3:**
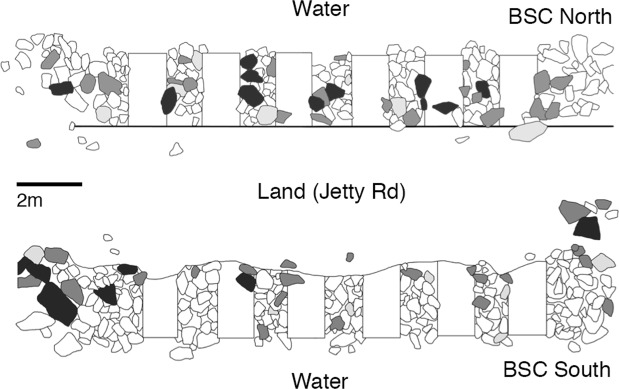
Plan of the Bennett Slough Culverts site. View from above, showing alternating pipes and piles of rocks (the width of Jetty Rd is reduced for conciseness). Darker shading on rocks indicates a higher use-intensity score.

Here, we report an archaeological and behavioral study of emergent anvil use by sea otters at the Bennett Slough Culverts (BSC) site near Moss Landing, California, USA. The site consists of six large metal drainage pipes surrounded by boulders, connecting two tidal wetland areas either side of a minor road (BSC North and BSC South; Figs 2 and 3; see Methods). We describe the behavior and physical outcomes of sea otter emergent anvil use to pound open mussels (*Mytilus* sp.), as an aid to future investigations into the geographical and historical spread (i.e. time-span, locations, and frequencies of occurrence) of this activity throughout the former sea otter range. Furthermore, for archaeologists who excavate past human behavior, it is crucial to be able to distinguish the evidence of sea otter food consumption from that of humans^13,14^. Our study establishes a new path for the growing field of animal archaeology, which until now has focused on primates^15–17^.

## Results

We report on two data sets, the first containing sporadic observations of sea otters foraging at the site from 2007 to 2017, and the second consisting of concentrated behavioral and archaeological observations at the BSC site from July 7^th^ to 27^th^, 2016. Because not all otters that used the site could be individually identified, there may be data on some of the same individuals in the two datasets. We did not collect foraging data during the ethoarchaeological observations in 2016, when we concentrated on obtaining video footage of sea otters pounding mussels on emergent rocks, describing the resulting wear patterns on the rocks, and documenting the characteristic distributions and fracture patterns of the broken mussel shells that accumulated below the rocks.

### Sea otter foraging observations at BSC 2007–2017

BSC is not a main foraging area for most sea otters in Elkhorn Slough (Fig. 2); however, small numbers of individuals have been observed in the area sporadically since 1998. We opportunistically observed tagged (see Methods) and untagged individuals foraging at BSC, with observation effort varying over time, and records spanning from 2007 to 2017. Observation effort at BSC was recorded only when otters were present and foraging. The increasing number of observations over time in our dataset reflects the increasing number of otters using BSC to forage as population numbers grew^18^. Beginning in 2007, we occasionally observed five tagged females feeding at BSC and in 2013 we began documenting foraging behaviors of untagged otters using the site as well. Foraging data from untagged individuals represented a minimum of four individuals (1 male and 3 females), but potentially up to 17 unique individuals (if each forage bout was from a different individual). In total, we recorded 629 dives from 29 forage bouts (Supplementary Data 1).

Mussels were the most common prey consumed by otters at BSC, comprising 51.2% of prey captures, followed by clams (25.4%), crabs (17%), and all other prey items (6.4%) (Supplementary Data 1; prey were not always identifiable to genus or species level). The average shell length of consumed mussels was 5.6 cm (±2.2 cm, n = 322), and the average rate of biomass intake was 6.9 grams per minute, about 25 to 75 mussels per hour (calculated from the size and number of mussels consumed and the relationship between shell length and edible biomass; see refs. ^19,20^). An analysis of variance showed that the proportion of mussels in the diet did not differ significantly between males and females (F_1,27_ = 1.05, p-value = 0.32).

All stone-use observed at BSC was performed by female otters; however, more females than males used the site. Emergent anvil use was only observed with the consumption of mussels, and was observed in 13.8% (4 of 29) of forage bouts. Within these four bouts, emergent anvil use occurred in 79.1% of mussel captures (n = 69, SE = 20.9%), meaning that 21% of individual mussels gathered by otters were pounded on emergent anvils. In addition to emergent anvils, otters also used other mussels and empty shells as chest anvils, and we observed an otter pound a Washington clam (*Saxidomus nuttalli*) on a stone chest anvil. While sea otters were observed foraging at BSC during all tide levels (−0.2 to 5.6 feet), emergent anvil use was only recorded during mid-to-high tide levels (2.1 to 5.4 feet).

### Ethoarchaeological study

During our ethoarchaeological study in July 2016, two tagged females with pups, three or more untagged females, and one untagged male visited the BSC site. Two of the female otters used emergent anvils, and a third female used a stone anvil on her chest. Emergent rocks were used as anvils, and one otter once used the side of a metal pipe culvert. Each otter began foraging by collecting multiple, clumped mussels during a foraging dive, usually from inside the pipes. When the otter returned to the surface, it held the clump securely on its chest and in the folds of its fur, while opening each mussel in turn with its teeth or a stone.

All observed emergent anvil use during this part of the study was mussel-pounding by two adult female sea otters (Supplementary Data 2), one of which is also listed in the foraging data collected from 2007 to 2017 (Supplementary Data 1). These sea otters held a mussel between both fore-paws and struck it rapidly and repeatedly against a rock (Video 1). Each otter would position itself either (i) floating on its stomach, side, or back, with its head and fore-paws upright (otters can achieve this posture because their skin is very loose on their body), holding the mussel up and striking downwards (Fig. 1B), or (ii) floating on its side, with the mussel then struck sideways or slightly upwards against an emergent anvil (Video 1). We observed both postures when the otters were striking at submerged anvils (Supplementary Fig. 1; Video 2), but for striking anvils at or above the water line the former was much more common (31 downward striking postures and 3 sideways striking postures; Supplementary Data 2). In each instance, the mussel was held compressed between the paws, so that each paw pushed against one of the mussel’s shells or valves. The otter continued striking until the shell was sufficiently weakened to allow the otter to use its teeth to lever the mussel open. Once a mussel was opened, the otter consumed the meat while floating on its back at the surface, close to the emergent anvil. While the sea otter ate, it performed cleaning rolls that allowed shell pieces to fall from its chest to the Slough bed (Video 3).

We coded 60 pounding series (each series involves a single, consumed mussel) from our video recordings of two adult females using emergent anvils at BSC (Supplementary Data 2). One otter struck anvils on both ridges and points above water, with a preference for points (16 of 20 series). The second otter struck only on ridges, both above and below water. Neither otter struck on rock faces. For classification of rock surfaces into ridges, points and faces, see Methods.

### Use-damage on rocks at BSC

**Figure 4:**
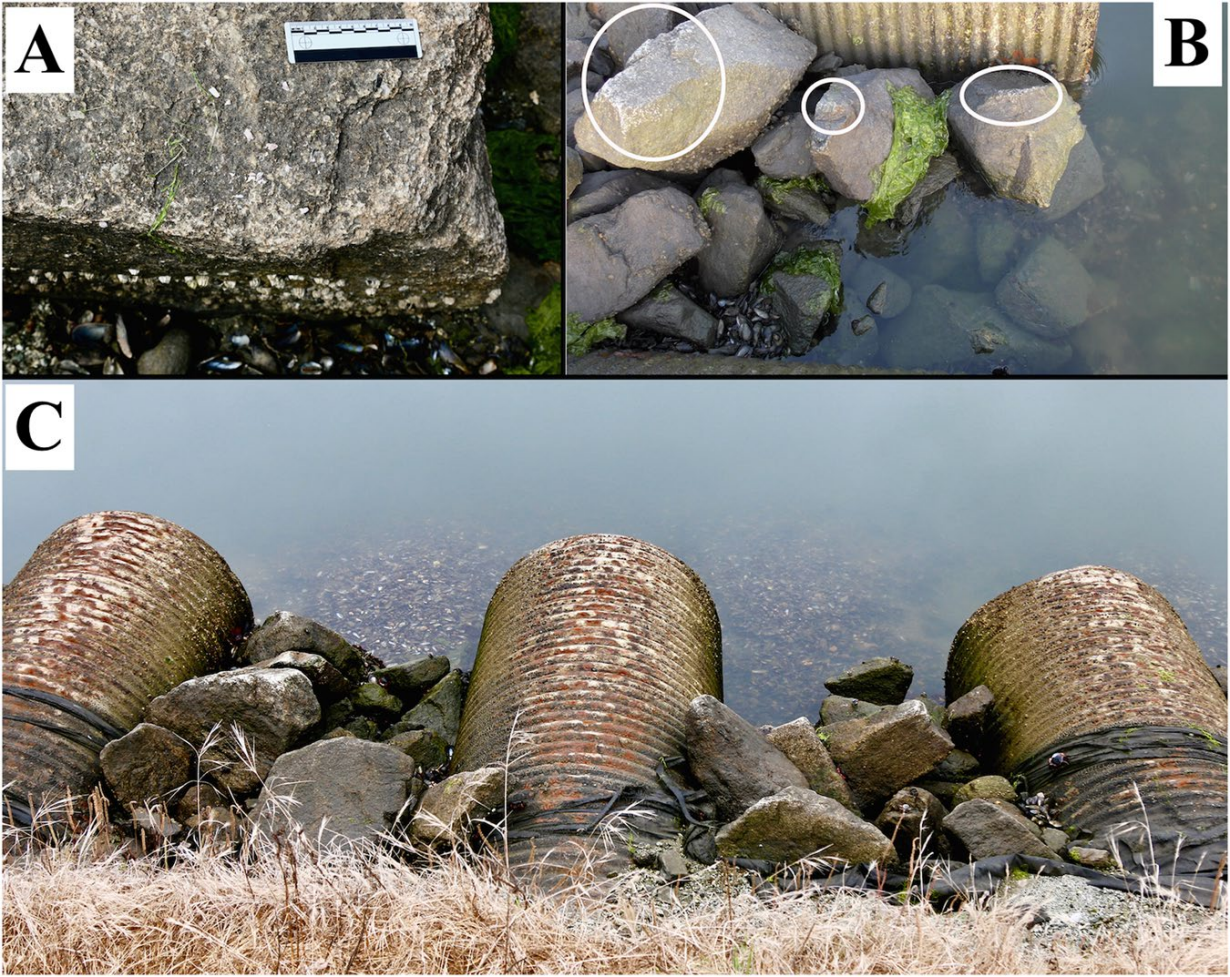
Use-wear damage on rocks at the Bennett Slough Culverts (BSC) site. (**A**) Sea otter damage on the corner of a quartzite boulder at BSC South; the scale is 10 cm. (**B**) Emergent boulders damaged by sea otters on their upper surfaces (circled) at BSC North, with the rocks further from the water topographically higher; the water level is mid-height. (**C**) Emergent anvils at low tide at BSC North, with the boulders seen in (**B**) on the left. Mussel shell deposits are visible above and below water in the three views.

In total we mapped 421 rocks. We followed standard protocols, such as those used on prehistoric anvils^21^, to assess sea otter pounding use-damage. Of the 421 rocks mapped at the site, 419 were quartzite and two were concrete blocks. Seventy-seven rocks (18.3%) had macroscopically visible use-wear in the form of crushed and fractured quartz grains on abraded ridges and points, exposing visibly lighter quartz grains (Figs 3 and 4). These damaged rock ridges and points included the ones pounded by otters during our ethoarchaeological observations (Video 2). For classification of emergent anvil use-damage see the Methods. There was no significant difference in the percentage of use-worn rocks at BSC North and BSC South (χ^2^ (1, *N* = 421) = 1.68, p = 0.195) (Table 1); therefore we combined all rocks into our use-wear analysis.

**Table 1:** Use-zones and total use-damaged anvils by location, Bennett Slough Culverts (BSC) site. Note that one anvil may have multiple used zones, so the number of use-zones exceeds that of anvils.

Use-zone	BSC North	BSC South	Total
Highest surface	40	35	75
Upper half, facing water	26	24	50
Lower half, facing water	0	3	3
Upper half, landward	0	2	2
Lower half, landward	0	0	0
Total use-zones	66	64	130
Total anvils	40	37	77
Total undamaged	148	196	344
Total rocks at BSC	188	233	421

Points were significantly more damaged than ridges on the highest part of a rock, while ridges were more damaged than points on an anvil’s upper half (for division of rock surfaces into use-zones, see Methods) (χ^2^ (1, *N* = 126) = 25.532, p < 0.0001) (Table 2). Flat faces were not damaged by pounding at BSC. Thus, otters preferentially target points and ridges as pounding surfaces. The intensity of wear was not significantly different between the highest surfaces and upper, water-facing parts of the emergent anvils (two-tailed Mann-Whitney test, U = 1558.5, z = 1.59, p = 0.112). No rock had use-damage on its lower land-facing side, and only two had wear on the upper land-facing side, both on ridges. Similarly, only three rocks had use-wear on the lower water-facing side. In contrast, 50 rocks had use-damage on their upper half facing the water, and 75 on the highest point of the rock. Forty-eight of the 50 anvils (98%) with water-facing damage on their upper half also had use-wear on their uppermost surface. These use-wear patterns indicate that the pounding damage occurs from otters pounding downward onto the upper parts of anvils, from a position in the water. Consistent with our long-term data set finding that emergent anvil use by sea otters was only recorded during mid-to-high tide levels, we found that rocks closer to land had more intense use-damage. Just under a quarter (23.4%) of damaged anvils had a high use-intensity score, and indicate sea otters repeatedly target landward rocks (Table 3), which are topographically higher than rocks closer to the water and thus spend more time exposed above water throughout the tidal cycle.

**Table 2:** Frequency of damaged use-zones with a given morphology, Bennett Slough Culverts site.

Use-zone	Points	Ridges	Faces	Total
Highest surface	51	22	2	75
Upper half, facing water	11	38	1	50
Lower half, facing water	1	1	1	3
Upper half, landward	0	2	0	2
Lower half, landward	0	0	0	0
Total	63	63	4	130

**Table 3:** Frequency of damaged anvils with a given use-wear intensity at the Bennett Slough Culverts site.

Intensity	BSC North		BSC South		Total
	Water-facing	Landward	Water-facing	Landward	
High	5	6	1	6	18
Medium	11	11	5	18	45
Low	3	4	3	4	14
Total	19	21	9	28	77
% High	26.32	28.57	11.11	21.43	23.38

### Shell debris at BSC

**Figure 5:**
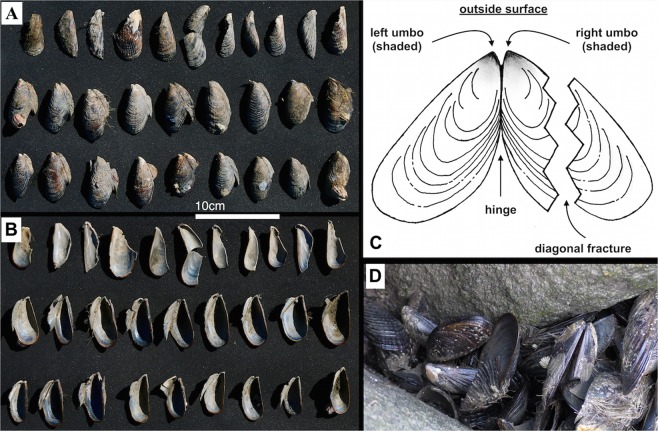
Mussel shell breakage patterns at the Bennett Slough Culverts site. (**A**) Outer and (**B**) inner faces of each valve; (**C**) schematic drawing of the exterior of a mussel shell showing the typical sea otter breakage pattern (illustration by Neil Smith); (**D**) broken mussel shells *in situ*.

During mussel pounding, the sea otters appear to have struck the right valve preferentially against the emergent anvils. This process resulted in the valves remaining attached to each other, with the right valve damaged or missing, and the left valve with an undamaged hinge (Fig. 5). Of 29 randomly sampled shell fragments, 18 were intact left valves with the hinge intact, and 11 were fractured right valves with no hinge. Thus, 100% of intact valves were left valves, and 100% of broken valves were right valves: the probabilities of these outcomes under a null hypothesis of equal likelihood of fracture for right or left valves are 0.000004 and 0.0005, respectively. The undamaged valves did not retain stone impact marks, and had an intact hinge and umbo, the protruding anterior part of the mussel shell (Fig. 5). The damaged right valves (both the sections still attached to undamaged left valves and the pieces removed by the otter) displayed radiating fractures, with a dominant fracture running diagonally up the shell.

There are dense shell middens around BSC, most visibly on the north side, that remain underwater at all tidal heights (Supplementary Fig. 2). These enhydragenic (sea otter-derived) middens appear to be thickest around the emergent anvils between the culvert pipes, and thinnest at the culvert pipe outlets (Fig. 4C). To avoid disturbing the otters we did not excavate the middens, but by extrapolation from surface counts we estimate that there are tens to hundreds of thousands of shells present. There are also dense mussel deposits that remain despite tidal incursions at the base of heavily used anvils where surrounding rocks trap the shells. The highest density that we counted was more than 100 shells within 30 cm of an anvil (Supplementary Fig. 2B). Of the 26 anvils with shell debris present within 30 cm, 22 (84.6%) had shells only on the water-facing side of the rock. All shells that were attributable to otter consumption were mussels, although other prey such as Washington clams and crabs (multiple genera of shore crabs and cancer crabs: Cancridae, Grapsidae, and *Pugettia* sp.) are present in the local environment and regularly consumed by the otters.

## Discussion

By combining behavioral observations and archaeological survey methods, we show that sea otters have created a distinct, recognizable archaeological record at the BSC site that allows interpretation of the behaviors that take place there. Our observations demonstrate that sea otters use emergent anvils specifically to open mussels, and that individuals use this strategy repeatedly within a single forage bout. Additionally, the long-term foraging observations show that this behavior has been occurring at BSC for at least 10 years. The use-damaged rocks and accompanying shell debris accumulations at the site form an unambiguous behavioral signature that can be used to identify other sites of emergent anvil use and contribute to our understanding of former sea otter foraging areas.

The use of emergent anvils only by female otters consuming mussels adds new data to previous sea otter stone-use and tool-use studies^3,4^. The mussel bias observed may be because otters collected mussels from within the culvert pipes, and thus were very close to shore making emergent anvils convenient pounding substrates. In contrast, Washington clams consumed at BSC were retrieved in deeper water where otters would be more likely to find a portable rock or shell to use as a chest anvil without needing to swim closer to shore.

Although we observed both sexes foraging on mussels at BSC, only females were seen using emergent anvils. Males only opened the mussels with their teeth and some females also opened mussels with their teeth (Supplementary Data 1). During our ethoarchaeological monitoring, we noted that two females with dependent pups swam away from shore to consume mussels after diving for mussels in the BSC pipes. One of these females occasionally used a chest anvil. As the BSC site was constantly visited by tourists and photographers on most of our monitoring days, Jetty Road was frequently lined with humans watching otters. It is possible that the females who swam away did not use the emergent anvils because they were uncomfortable with the proximity of humans. Furthermore, females that used emergent anvils also opened mussels with their teeth, suggesting that anvil pounding is not motivated by sex differences in jaw strength. While a slight female bias towards stone use has been shown in previous studies^4^; the lack of male anvil use here may also result from our small sample size. Although observations on males were conducted from 2014 to 2017 at BSC, it is possible that we observed the same male each time, one which did not use stones.

We found that wild sea otters prefer to break open mussels on emergent anvils that have points and ridges on their upper, water-facing parts. Concentration of use-wear on the water-facing upper surfaces of the rocks demonstrates that the damage is not caused by incidental contact with floating debris, which would evenly affect the upper and lower rock surfaces, and is clearly distinct from anthropogenic impacts, which would cause scuffing or smoothing of upper, land-facing rock surfaces. Similarly, the focus on protruding points and ridges on the water-facing upper parts of rocks is diagnostic and consistent with observed sea otter behavior.

Characteristic use-damage patterns on rocks and shells, together with extensive underwater midden deposits, have not been previously reported for marine mammals, although shell middens created on land by river otters without the use of tools or pounding have been documented^13,14,22^. A combination of specific environmental factors is required to produce sea otter middens, including a low-energy depositional environment such as an inlet, slough or estuary, abundant hard-shelled prey, and the presence of suitable hard surfaces for use as anvils. BSC provides an ideal microhabitat for mussel settlement and growth, due to the oxygen and nutrients brought by the daily incoming and outgoing tides, and can support high rates of sea otter feeding.

Our study links sea otter behavior to its archaeological record in the form of damaged rocks and large shell middens. We estimated the rate at which mussel shells might accumulate near the emergent anvils. If just one otter visits BSC for one hour per day (the mean length of a sea otter foraging bout^23^) to feed on 25 to 75 mussels (Supplementary Data 1), and if 21% of those mussels are pounded on the emergent anvils, then 1,916 to 5,748 mussels are pounded by otters at BSC annually. This figure is very conservative, given that foraging occurs with similar frequency throughout the day and night^24,25^, and adult otters spend 35–55% of their time feeding^23,26^. If sea otters started using the BSC boulders as soon as they recolonized the area in 1994, then in 23 years of site use, we would expect a minimum of 44,073 to 132,221 mussels to have been pounded on emergent anvils at BSC. This figure appears to be compatible with the size of the underwater mussel deposits we observed at the site (Supplementary Fig. 2A), but excavation would be necessary to test this prediction.

A selection of shells collected from the middens showed a consistent shell breakage pattern that is characteristic of predator action^27^: the combination of an intact left valve still attached by the hinge to a fragment of the right valve, and separate fragments of right valves with diagonal fractures. This breakage pattern is the same as on *Saxidomus* clams broken by sea otters^27,28^, and is distinct from that of mussels opened by sea otters with their teeth, which consists of pairs of umbos attached by the hinge, valves missing their umbos, and small angular shell fragments^29^. It is also different from river otter debris, which consists of small fragments of digested mussel shells deposited in spraints (otter feces) at rock shelters^14^. In combination with the large mussel shell middens at BSC, the shell breakage patterns provide a novel way to distinguish mussels broken by sea otter pounding on emergent anvils from those broken by humans or other animals.

The absolute lateralization of shell breakage patterns from emergent anvil use is also unusual, and strongly supports a lateral preference in the way the otters handle the mussels. This breakage pattern is not due to an asymmetric ‘weak spot’ on the right valve of these mussels, as there is no difference in shell strength of right and left valves in the mussel species found at BSC, as measured by point-loading experiments^30^. Nor is the breakage pattern caused by fluctuating asymmetry due to thermal stress or environmental pollution^31,32^, because in this case we would expect to find equal numbers of right and left broken shells. Instead, the observed bias is more consistent with lateralized handling by otters. We speculate that the sea otter(s) that produced the deposit we sampled from held mussels preferentially with the right valve’s umbo slightly oriented toward the anvil, perhaps due to pawedness. Lateralized behaviors occur in a variety of mammals^33–35^, including the Asian small-clawed otter^36^, and upper limb laterality is expressed more strongly with skilled manipulations, precision handling, pounding, or tool-use in primates^37,38^. To begin to explore this hypothesis, we post-hoc analysed the 60 pounding events by viewing our videos frame-by-frame. Our sample (Supplementary Data 2) had 18 events with a clear view of pounding above water with the otter’s paws, mussel, and rock visible, from which we could potentially determine the mussel’s position as it contacted the rock. Four of these pounding events show the paws symmetrically positioned on either side of the mussel. However, in 14 events (7 by Red-Pink and 7 by ChocChip), there is a clear lateralised handling. The otters appear to turn the wrist just before impact so that the right paw is oriented palm down on top of the mussel and the mussel’s right valve hits the rock. This positioning contrasts with the mussel and paw orientation during the downward phase of the forearms and the upward phase in preparation for a strike, in which the otters hold the mussel symmetrically between their paws, with each paw pressing against one valve, and the shell margin oriented toward the rock. In further support of these observations, four of our video clips show the mussel’s right valve fracturing as a result of the pounding. These few data points suggest that otters are lateralised during pounding; thus we cannot reject the hypothesis that sea otter laterality is related to the characteristic asymmetrical shell breakage patterns we found. However, this hypothesis remains to be tested via targeted research on sea otter mussel pounding kinematics with more individuals and controlled filming conditions.

Our study shows that a clear archaeological signature of sea otter emergent anvil use can result from the behavior of a small number (10 to 20) of individuals over a few years. More broadly, the recovery of past animal behavioral traces helps us to understand the evolution of behaviors like stone anvil use, which is rare in the animal kingdom and is extremely rare in marine animals. In addition to sea otters, anvil use occurs in fishes of the family Labridae, which break open scallops and urchins held in the mouth on fixed underwater anvils by striking the prey against the anvil^39^. In addition, many bird species break open mollusks, nuts, or bones on stone anvils by dropping the food from the air^2^. We predict that these activities produce enduring use-wear on the anvils and accumulations of debris that can be studied to learn about behavioral evolution in a given species, for example in nut-dropping by crows^40–42^. Interdisciplinary approaches like the one presented in this study have proven successful in identifying food-pounding anvil sites that were historically used by apes and monkeys^5–7,9,15–17^, thus providing insights into long-term behavioral transmission and cultural evolution^43^. For instance, archaeological studies have shown that nut-cracking on stone anvils by bearded capuchins (*Sapajus libidinosus*) in Brazil extends at least 600 years into the past, representing over 100 generations of the behavior^16^, and locations that were clearly heavily used in the past are no longer in the monkeys’ foraging route^44^. Both use-damaged rocks and discarded mollusk shells are relatively long-lasting materials.

At BSC, the concentrated deposition of large amounts of shells with the specific breakage patterns in a low energy water environment, combined with anvil use-damage, suggests that emergent anvil use can be detected in locations previously inhabited by sea otters. This information could help to document past sea otter presence in locations where they are now extinct, in conjunction with other analyses such as analysis of sea otter bones found in human archaeological middens, radiocarbon dating, and size reconstructions of shells^45–47^. Fortunately, the rapid development of diagnostic indicators at BSC shows that new sites can be quickly occupied and altered by sea otter behavior, ultimately creating a distinctive archaeological record that parallels and may even pre-date that of the humans they currently live alongside.

## Materials and Methods

### Study site

The Bennett Slough Culverts (BSC) site is located approximately 100 m west of the junction of State Highway 1 and Jetty Road at Moss Landing, California (N3649′01″, W12147′14.5″) (Fig. 2). The current Bennett Slough Culverts were completed in 1991, following collapse of a previous structure in the 1989 Loma Prieta earthquake^48^. Sea otter use of the current site configuration therefore postdates 1991. The BSC site lies between Bennett Slough to the north and Moss Landing North Harbor to the south. To the southeast of BSC is Elkhorn Slough, a State Marine Conservation Area and State Marine Reserve, which became part of a freshwater river system feeding westward into the Monterey Canyon^49^ due to lowered sea levels during the last glacial maximum (about 18,000 to 16,000 years ago). The site geology is sedimentary, overlying Salinian Block granite, which does not outcrop near the BSC site. As the sea reached its present level in the early Holocene, the area evolved from a high-energy tidal inlet into an estuarine environment associated with the shifting mouth of the Salinas River system that met the ocean north of the present day BSC. Human occupation in the area has been dated back to 8,000 years before present (BP), with human hunting of sea otters documented from around 6,000 BP at Elkhorn Slough^50^. The present-day geographic configuration of local jetties created the Moss Landing Harbor in 1946–7^49^. This construction moved the mouth of the Salinas River and directly opened the Elkhorn Slough estuary to Monterey Bay, changing the estuary from a predominately fresh water system to a tidally influenced watershed, and the old mouth of the Salinas river (north of BSC) naturally filled with sediments^49^.

BSC consists of six large metal drainage pipes, each 1.25 m in diameter and just over 21 m long, that pass underneath Jetty Road in a north-south orientation. The pipe ends are exposed for approximately 2.3–2.5 m on either side of the road. The pipes are surrounded by mostly quartzite boulders, with a small number of concrete slabs, that form part of the construction. Boulders closer to the water are topographically lower. Highest tides cover the pipes and lowest tides expose the pipes completely. Unlike the pipes, the lowest boulders at the site are never fully exposed at low tide, and the highest boulders are never fully submerged. Due to the constantly flowing water supplying suitable nutrients, abundant mussels grow around and within the BSC pipes (Supplementary Fig. 3). Although tidal movements continuously determine the specific rocks that are present at the water line, and the time of day that a given rock is exposed, the cyclical nature of the tides ensures that all rocks in the intertidal zone are regularly available for potential use as anvils by sea otters.

#### Foraging observations

The present sea otter population began to recolonize Elkhorn Slough in 1994 after being historically extirpated due to hunting. Beginning in 1998, live-stranded sea otters that were raised at the Monterey Bay Aquarium (MBA) were re-released in Elkhorn Slough and monitored opportunistically by radio telemetry and visual observations to monitor survival and health^51,52^. These otters were tagged on their hind flippers with various combinations of colored cattle ear tags to enable visual identification of individuals^53^. Observations at BSC prior to 2013 were dependent on the presence of tagged individuals and thus occurred sporadically as most otters remained in other parts of Elkhorn Slough (Fig. 2). Since 2013, we collected data on sea otter foraging behaviors daily throughout Elkhorn Slough during daylight hours on both tagged and untagged individuals. However, BSC remained a feeding and resting area for only a few animals at any given time, so BSC was not the main site that yielded foraging data. Thus, data from BSC were collected from otters that mainly fed in other locations within the slough.

An otter was considered foraging when seen repeatedly diving for food and returning to the surface to manipulate and consume prey or to take a breath before diving again. A series of dives by a single individual was considered a forage bout, following standard methods^3^. The age, sex, and reproductive status (presence and approximate age of pup) were visually determined using standard methods^20^. Foraging data collection followed standard protocols used in previous studies^4,20,54^ and included the success of each dive, the identification of the prey item to the lowest taxonomic level possible, approximate prey size, and whether and how any tool-use or stone-use occurred (Supplementary Data 1). Although prey were identified to species level when possible, they were grouped into 5 functional groups or prey types by taxonomical or morphological similarity to address variation in taxonomic resolution. The frequency of emergent anvil use was calculated by bout and dive.

#### Ethoarchaeological monitoring

Ethoarchaeological monitoring at the BSC site took place over 20 days in July 2016 (Videos 1–3), during daylight hours and on the night of July 19^th^. We saw otters visit the site on 11 of these days. Because this was an exploratory study, we first conducted preliminary observations on the interactions between sea otters and the rocks and pipes at the site under various tidal conditions. These enabled us to identify the locations where sea otters were pounding and learn to identify the individuals using the site. We then carried out archaeological surveys on the locations (boulders) used by the otters. We used a Canon 70D digital camera to obtain photographs of the site and the otters. When conditions at the site were favorable (good light, little spray, no crowds of tourists or wildlife photographers standing over the otters causing noise and disturbance), we filmed the sea otters pounding. We used a Panasonic 4K video camera at 25 frames per second. Otters often visited the site without pounding, but we obtained good videos of two adult females (one tagged, one untagged) that repeatedly visited the site and broke open mussels by pounding on emergent anvils on July 7^th^ and 14^th^ 2016 (Supplementary Data 2). These recordings enabled us to identify which parts of the rocks these two otters used, the average number of strikes needed to break open a mussel, and the way in which the otters held the mussels while pounding. Following ref.^12^, we defined a pounding series as starting when a sea otter began pounding its prey and ending when the pounding stopped and consumption started. From the videos, we also noted the positioning of the prey item during pounding (Column I in Supplementary Data 2, labeled “Strike point on mussel”).

### Archaeological surveys

Because the pounded rocks are fixed at the shoreline, we predicted that recurrent instances of hard-shelled prey pounding would result in the development of use damage on the anvil surface, and the build-up of discarded shell fragments around the activity site. A similar pattern of debris build-up on land has been noted for North American river otter (*Lontra canadensis*, renamed from *Lutra canadensis* in 2015) shell middens in southeast Alaska^13,14,22^. To quantify the archaeological evidence, we mapped a 20 m segment of each part of the site (BSC North and BSC South) at low tide, perpendicular to and centered on the pipes (Fig. 3). We mapped all rocks greater than 30 cm in their maximum dimension in order to map only fixed anvils by excluding smaller rocks that could have been picked up by otters for use as chest anvils.

We recorded all presence and absence of use-damage on the rocks, dividing each rock surface into five zones for this purpose: (i) the highest surface, or topographically highest point on the rock; (ii) the upper half of the rock on the side facing the water (i.e., the north of the rock on the north side of the site, and vice versa); (iii) the lower half of the rock facing the water; (iv) the upper half of the rock facing the land (i.e., Jetty Road); and (v) the lower half of the rock facing the land (Figs 3 and 4). The damaged surfaces were recorded either as points (isolated, projecting sections of rock, often the intersection of multiple ridges), ridges (the elongated meeting of two rock faces), or faces (a relatively flat surface). Use-damage intensity was assessed on a scale of none, low, medium or high, with none being no visible damage, low being minor, isolated damage, medium being distinct but discontinuous wear, and high being extensive and continuous damage. Only the most intense damage on any given zone was recorded. As part of our assessment of sea otter spatial use of the site, we compared damage on rocks that are closer to the water (and topographically lower) to the more landward ones. To do so, we divided BSC North and BSC South rocks into two groups each (land-side vs. water-side), using the halfway point of the exposed pipes as a dividing line. We did not quantify damage on the metal pipes, as sea otters were never observed pounding on the tops of the pipes. The visible wear on the top surfaces of the pipes compared to the sides (Fig. 4) is likely of anthropogenic origin, as it is evenly distributed along the pipes’ entire length. BSC is a very popular wildlife-watching site, where the pipes and rocks are accessible to human contact. People repeatedly stand on the pipes or climb over rocks to get a closer view of otters and birds, and to take photographs. Anthropogenic impacts are more likely to appear as scuffing or smoothing of flat faces on rocks (as seen on boulders at the North/South Jetties in Moss Landing, where people frequently fish), but without causing fracturing or crushing damage.

We recorded the presence of shell debris extending 30 cm (the maximum distance that mussel fragments could be likely attributed to a particular rock) around each use-damaged rock, noting the number of shells and their position on either the water-facing side or land-facing side. We photographed, but could not directly access, shell deposits that lay beneath the water at the site. We recorded the breakage patterns on an opportunistic sample of 29 mussel shells from an exposed deposit at BSC South (Fig. 5; shown *in situ* in Supplementary Fig. 2B), which was the only accessible shell deposit. We selected only the topmost shells to minimize disturbance of the deposit for future excavations.

All sea otter research activity at the site was carried out under permits from the US Fish and Wildlife Service issued to M.T.T. and Monterey Bay Aquarium and with oversight by the Institutional Animal Care and Use Committee at the University of California Santa Cruz and Monterey Bay Aquarium. We only mapped and recorded the site when no otters were present, so that we did not interrupt feeding or other behaviors.

## Supplementary information

Supplementary Information

Video 1

Video 2

Video 3

Dataset 1

Dataset 2

## Data Availability

All data generated or analyzed during this study are included in this published article and its Supplementary Information files.
