# Supplementary material for: Wild sea otter mussel pounding leaves archaeological traces
Source: Sci Rep. 2019 Mar 14;9:4417. doi: 10.1038/s41598-019-39902-y (PMC6418163; doi:10.1038/s41598-019-39902-y)
Supplement: Supplementary file 1 — Supplementary Information [file 41598_2019_39902_MOESM1_ESM.pdf]

Supplementary Information

Wild sea otter mussel pounding leaves archaeological traces

Michael Haslam[1], Jessica Fujii[2]*, Sarah Espinosa[3], Karl Mayer[2], Katherine Ralls[4], M. Tim Tinker[3,5], Natalie Uomini[6]*

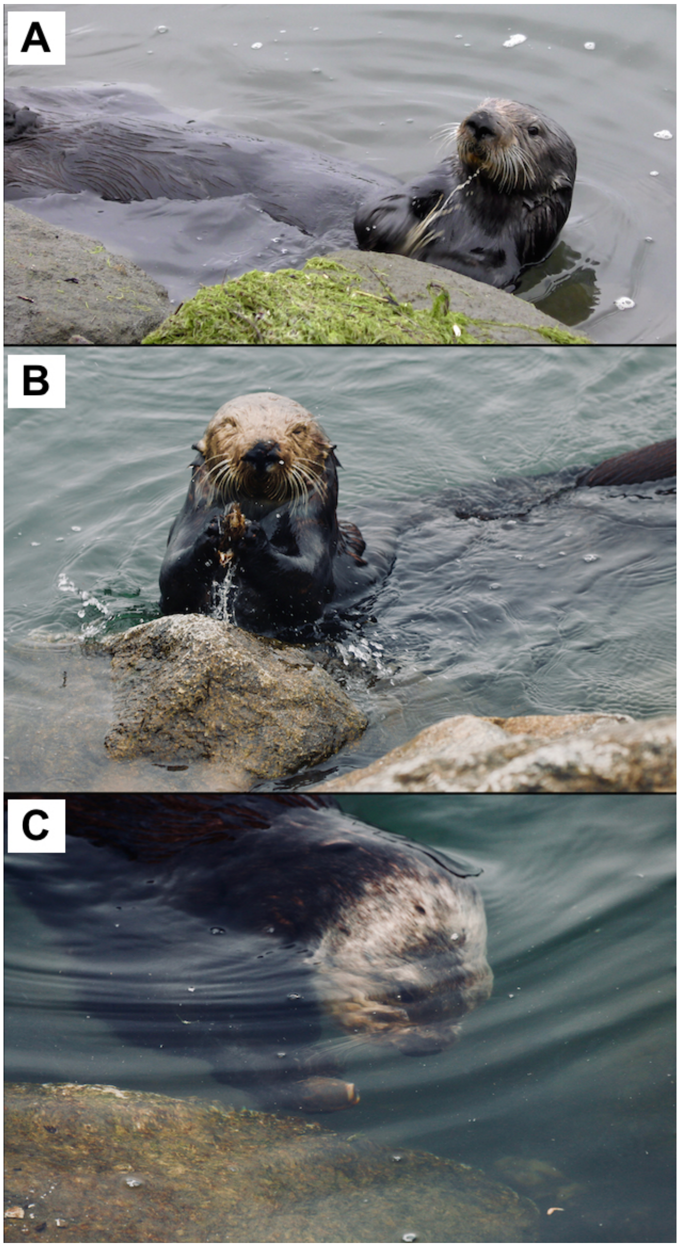

Supplementary Figure 1. Sea otters pounding open mussels at Bennett Slough Culverts. (A, B) Pounding above water, in sideways and upright position. Note the lighter-colored use-wear on the top of the emergent anvil in (B). (C) Pounding underwater, in sideways position.

Supplementary Information

Wild sea otter mussel pounding leaves archaeological traces

Michael Haslam[1], Jessica Fujii[2]*, Sarah Espinosa[3], Karl Mayer[2], Katherine Ralls[4], M. Tim Tinker[3,5], Natalie Uomini[6]*

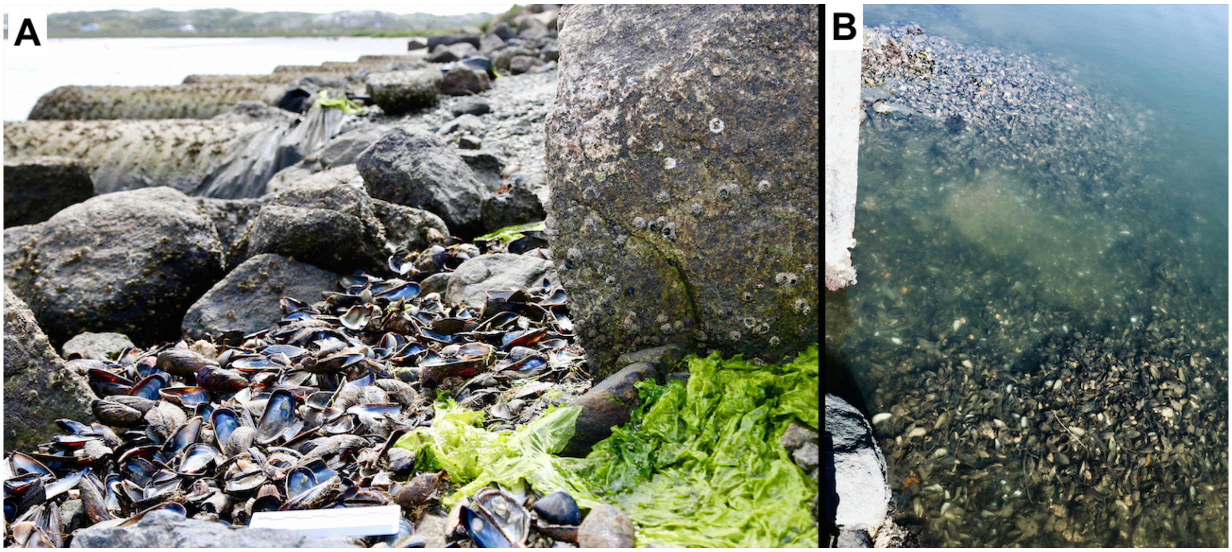

Supplementary Figure 2. Sea otter-generated mussel deposits at Bennett Slough Culverts (BSC). (A) Shell midden exposed on land, BSC South and (B) Underwater shell middens at low tide, BSC North.

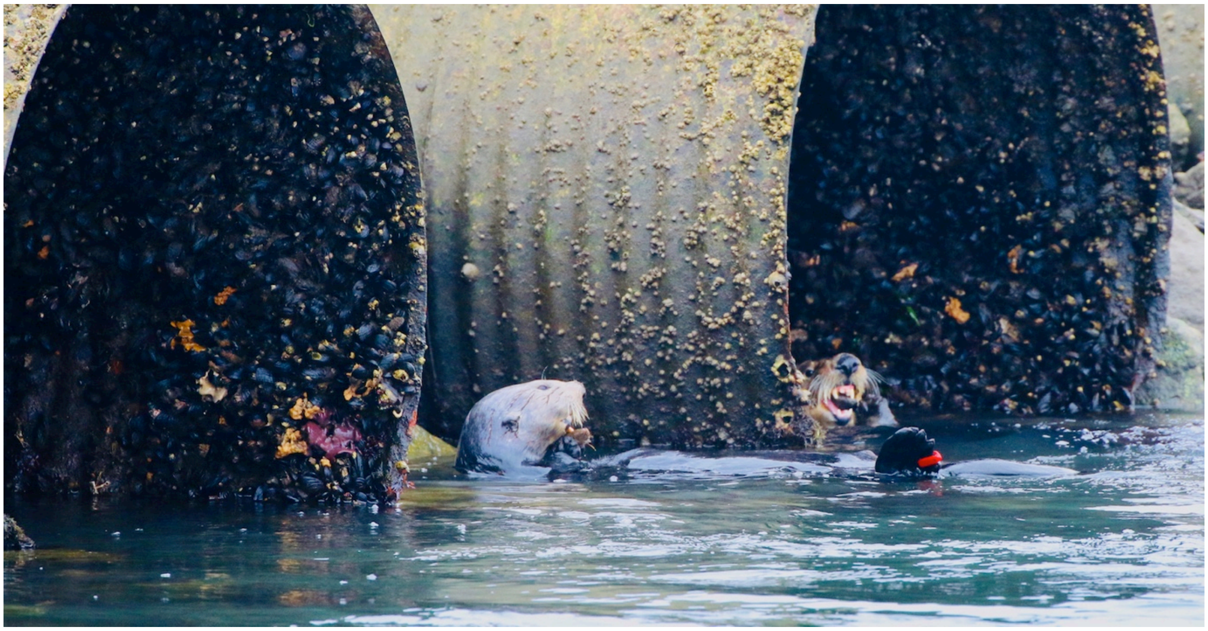

Supplementary Figure 3. Mussels growing in the pipes at Bennett Slough Culverts. The water level is close to low tide.

Supplementary Information

Wild sea otter mussel pounding leaves archaeological traces

Michael Haslam[1], Jessica Fujii[2]*, Sarah Espinosa[3], Karl Mayer[2], Katherine Ralls[4], M. Tim Tinker[3,5], Natalie Uomini[6]*

Video 1. Sea otter pounding a mussel on an emergent anvil, above water, at Bennett Slough Culverts. The otter is Red-Pink, code MBA 457-09.

Video 2. Sea otter at Bennett Slough Culverts biting, scooping with paws and eating mussel, discarding mussel shells, and pounding underwater with both sideways and upright postures. The otter is untagged, nicknamed ChocChip. Damage is visible on the anvil.

Video 3. Sea otter performing chest rolls while eating at Bennett Slough Culverts. Mussel shell breakage patterns are visible.

Data 1. Excel spreadsheet of the observed foraging behaviors of sea otters at Bennett Slough Culverts, 2007-2017. Metadata are listed in the spreadsheet.

Data 2. Excel spreadsheet of observed strikes per mussel on emergent anvils for two otters at Bennett Slough Culverts, July 2016. Metadata are listed in the spreadsheet.
